# The Malarial Exported PFA0660w Is an Hsp40 Co-Chaperone of PfHsp70-x

**DOI:** 10.1371/journal.pone.0148517

**Published:** 2016-02-04

**Authors:** Michael O. Daniyan, Aileen Boshoff, Earl Prinsloo, Eva-Rachele Pesce, Gregory L. Blatch

**Affiliations:** 1 Biomedical Biotechnology Research Unit, Department of Biochemistry and Microbiology, Rhodes University, Grahamstown, South Africa; 2 Biotechnology Innovation Centre, Rhodes University, Grahamstown, South Africa; 3 College of Health and Biomedicine, Victoria University, Melbourne, Victoria, Australia; 4 Department of Pharmacology, Faculty of Pharmacy, Obafemi Awolowo University, Ile-Ife, Nigeria; UMCG, NETHERLANDS

## Abstract

*Plasmodium falciparum*, the human pathogen responsible for the most dangerous malaria infection, survives and develops in mature erythrocytes through the export of proteins needed for remodelling of the host cell. Molecular chaperones of the heat shock protein (Hsp) family are prominent members of the exportome, including a number of Hsp40s and a Hsp70. PFA0660w, a type II Hsp40, has been shown to be exported and possibly form a complex with PfHsp70-x in the infected erythrocyte cytosol. However, the chaperone properties of PFA0660w and its interaction with human and parasite Hsp70s are yet to be investigated. Recombinant PFA0660w was found to exist as a monomer in solution, and was able to significantly stimulate the ATPase activity of PfHsp70-x but not that of a second plasmodial Hsp70 (PfHsp70-1) or a human Hsp70 (HSPA1A), indicating a potential specific functional partnership with PfHsp70-x. Protein binding studies in the presence and absence of ATP suggested that the interaction of PFA0660w with PfHsp70-x most likely represented a co-chaperone/chaperone interaction. Also, PFA0660w alone produced a concentration-dependent suppression of rhodanese aggregation, demonstrating its chaperone properties. Overall, we have provided the first biochemical evidence for the possible role of PFA0660w as a chaperone and as co-chaperone of PfHsp70-x. We propose that these chaperones boost the chaperone power of the infected erythrocyte, enabling successful protein trafficking and folding, and thereby making a fundamental contribution to the pathology of malaria.

## Introduction

*Plasmodium falciparum* is the protozoan parasite responsible for the most virulent form of human malaria [1]. Although preventable and often curable, malaria remains a life-threatening disease with high mortality [2–4]. Despite the use of preventative approaches such as insecticide treated nets and indoor residual spraying [5,6], chemotherapeutic intervention is still necessary to the effort to eradicate malaria. However, the ability of *P*. *falciparum* to develop drug resistance [7,8] has made the search for new pharmacotherapy an imperative.

*P*. *falciparum* exports parasite-encoded proteins involved in structural and functional remodeling of the host cell. This process is essential for the development of the parasite and is associated with the pathology of the infection [9,10]. Among the exported proteins are heat shock proteins, functioning as molecular chaperones that are proposed to be highly adapted to the malaria parasite lifecycle [11]. The heat shock protein 70 (Hsp70) chaperone and its co-chaperone heat shock protein 40 (Hsp40) are involved in facilitating protein folding, stabilization, degradation, and translocation across membranes [12,13]. Hsp70s bind to short hydrophobic regions of unfolded substrate proteins in an ATP-controlled manner that is regulated by Hsp40 co-chaperones [14]. Hsp40s are characterized by the presence of the J domain needed for the stimulation of the ATPase activity of Hsp70s [15]. On the basis of their domains, the Hsp40s have been classified into types I-IV [16,17], with types I and II capable of binding to unfolded substrate proteins for targeting to partner Hsp70s [18,19]. There are six *P*. *falciparum* Hsp70s (PfHsp70s), five of which are parasite-resident, with PfHsp70-1 being the most well characterized [20–28]. PfHsp70-x is the only Hsp70 found in the parasitophorous vacuole (PV) and the infected erythrocyte cytosol [29,30]. The host cell cytosol contains residual human Hsp70 [31], and so it is tempting to speculate that PfHsp70-x may boost the chaperone power of this compartment to aid proper folding of the large exportome. PfHsp70-x has been shown to co-localize with two exported type II Hsp40s, PFE0055c and PFA0660w, in structures called J-dots in the infected erythrocyte cytosol. Furthermore, the J-dots associate with *P*. *falciparum* erythrocytes membrane protein 1 (PfEMP1), the major malaria virulence factor [29,32]. It has been proposed that PfHsp70-x/PFE0055c/PFA0660w play an important role in the trafficking and folding of exported proteins, including malaria pathogenesis factors [33].

Small-molecule inhibitor studies [34] and homology modelling [35] have been conducted on PfHsp70-x. However, the biochemical details of its interaction with exported plasmodial Hsp40s (PfHsp40s) remain to be elucidated. Attempts to obtain a viable *PFA0660w*-knock-out parasite line were unsuccessful, suggesting that this PfHsp40 may be essential to the parasite [36]. Although PFA0660w and PfHsp70-x co-localize in the J-dots [29], there is still no evidence for a direct or functional interaction. The high number of potentially exported Hsp40s together with the exported PfHsp70-x underlines the importance of the Hsp70/Hsp40 interface for the survival and development of the parasite within the erythrocyte [37,38]. Therefore, the experimental validation of a productive associate between PfHsp70-x and PFA0660w may further our understanding of their roles and pave the way for exploring this partnership in drug discovery.

In this work, recombinant PFA0660w was biochemically characterized to understand its functions alone and in combination with three Hsp70s: PfHsp70-1, PfHsp70-x and human Hsp70 (HSPA1A; [39]). Overall, we have presented the first biochemical evidence for a direct and potentially specific interaction between the exported molecular chaperones PFA0660w and PfHsp70-x.

## Materials and Methods

### *pQE30-PFA0660w* expression vector

The optimized coding sequence for expression of PFA0660w in *Escherichia coli* was synthesized and supplied by GenScript^(R)^ (USA). The PFA0660w coding region was inserted into the pQE30 expression vector (Qiagen, Germany) to produce a plasmid encoding the (His)_6_-PFA0660w (PFA0660w) protein.

### Heterologous expression and purification of PFA0660w

PFA0660w was over-expressed and purified using the *E*. *coli* M15[pREP4] strain (Qiagen, Germany). Protein expression was induced by addition of 0.4 mM IPTG (isopropyl-β-D-thiogalactopyranoside). Bacteria cells expressing PFA0660w were harvested by centrifugation and the cell pellet resuspended in lysis buffer (LB: 10 mM Tris-HCl, pH 8.5, 300 mM NaCl, 50 mM imidazole, 1 mM PMSF, 1 mg/ml lysozyme), allowed to stand for 20 min at room temperature and then frozen at -80°C overnight. Following thawing and sonication at 4°C, the insoluble pellet was washed three times in wash buffer (WB: 50 mM Tris-HCl pH 8.5, 200 mM NaCl, 10 mM EDTA, 1% Triton X-100, 1 mM PMSF) and twice in double distilled water as previously described [40,41]. The pellet was recovered after each wash by centrifugation (10000 × *g* at 4°C for 10 min). The pellet was then resuspended in solubilising buffer (SB: 100 mM Tris-HCl pH 8.5, 300 mM NaCl, 8 M urea, 50 mM imidazole, 5 mM DTT, 0.1 mM EDTA, 1 mM PMSF) and clarified by centrifugation at 16000 × *g* for 30 min at 4°C. To ensure proper refolding, the solubilised protein was diluted to a final concentration of 250 μg/ml with refolding buffer (RB: 100 mM Tris-HCl pH 8.5, 300 mM NaCl, 50 mM imidazole, 10% glycerol, 5% sucrose, 1 mM DTT, 0.1 mM EDTA, 0.1% PEG 2000, 1 mM PMSF) supplemented with 2 M urea and incubated with gentle stirring at 4°C for 2 h. The supernatant was filtered through 0.45 μm filters and loaded onto a 5 ml HisTrap HP column (GE Healthcare, UK). The column was washed with five column volumes of RB, followed by five column volumes of RB without PEG 2000. Protein was eluted with three column volumes of elution buffer (EB: 100 mM Tris-HCl pH 8.5, 300 mM NaCl, 0.5 M imidazole, 10% glycerol, 5% sucrose, 1 mM DTT, 0.1 mM EDTA, 1 mM PMSF). Mouse monoclonal anti-His antibody (1:5000; GE Healthcare, UK), mouse monoclonal anti-DnaK antibody (1:5000; Sigma–Aldrich, Germany) and anti-mouse HRP-conjugated secondary antibody (1:10000; GE Healthcare, UK) were used to confirm the presence of the target protein and rule out the presence of contaminating DnaK (*E*. *coli* Hsp70). The purified protein was stored at -80°C or dialysed into dialysis buffer (DB: 50 mM Tris-HCl pH 8.5, 300 mM NaCl, 1 mM DTT, 1 mM PMSF, except otherwise stated) for further studies. Secondary structure analysis of the refolded protein was performed using Fourier transformed infrared (FTIR) spectroscopy as previously described [42].

### Purification of Hsj1a, PfHsp70-1, PfHsp70-x and HSPA1A

Recombinant (His)_6_-Hsj1a (Hsj1a is formally called DNAJB2a), (His)_6_-PfHsp70-1 and (His)_6_-PfHsp70-x (Hsj1a, PfHsp70-1, PfHsp70-x) were purified under native conditions as previously described [24,34,43], while recombinant HSPA1A-(His)_6_ (HSPA1A) was purified according to Chiang and colleagues [23]. Samples were dialysed into DB or into the appropriate assay buffer for functional studies.

### Size exclusion chromatography of PFA0660w

Size exclusion chromatography of purified PFA0660w was performed using an ÄKTAbasic FPLC system with a Superdex 200 HR 16/60 column (Amersham Pharmacia Biotech, UK), equilibrated with a suitable buffer (50 mM Tris–HCl, pH 8.5 and 0.15 M NaCl). The following molecular mass standards were used: blue dextran (2000 kDa—void volume), catalase (240 kDa), BSA (66 kDa), ovalbumin (45 kDa), carbonic anhydrase (29 kDa), RNase A (13.7 kDa) and lysozyme (14.3 kDa). The flow rate was maintained at 1 ml/min.

### ATPase assays

The ability of PFA0660w to stimulate Hsp70 ATPase activity was determined by colorimetric assay as previously reported [26,34] with modifications in the ATPase buffer composition as follows: 50 mM Tris-HCl pH 8.5, 2 mM MgCl_2_, 100 mM KCl, 0.5 mM DTT. Submolar (0.2 μM), equimolar (0.4 μM) and molar excess (0.8 μM) concentrations of the Hsp40 to 0.4 μM of the Hsp70s (PfHsp70-1, PfHsp70-x, HSPA1A) were used. All assays were corrected for spontaneous ATP hydrolysis and inactivated Hsp70s (boiled for 15 min at 100°C) were used as controls. Assays were conducted in triplicate, and at least three separate experiments were performed using batches of independently purified proteins. Specific basal ATPase activity was expressed as nmol Pi released/min/mg Hsp70 protein and percent fold increase. Data are presented as mean ± standard error of mean (mean ± SEM).

### Aggregation suppression assay

The capacity of PFA0660w to suppress the aggregation of rhodanese, a model protein for aggregation studies, either alone or in the presence of PfHsp70-1, PfHsp70-x or HSPA1A was adapted from previously published methods [44–46]. Briefly, aliquots of 300 μM bovine rhodanese (Sigma-Aldrich, Germany) in 50 mM Tris-HCl pH 8.5 were denatured for 3 h at 25°C in denaturing buffer (6 M guanidine hydrochloride, 50 mM Tris-HCl pH 8.5, 300 mM NaCl, 1 mM DTT) to a concentration of 25 μM. Denatured rhodanese was diluted into reaction buffer (50 mM Tris-HCl pH 8.5, 300 mM NaCl, 1 mM DTT) to a final concentration of 1.5 μM before the rate of its aggregation was monitored at 320 nm for 20 min at room temperature with a Helios Alpha UV-Vis spectrophotometer (Thermo Scientific, USA). Varying concentrations of PFA0660w or each Hsp70 in combination with the Hsp40 were added to the assay buffer and equilibrated at room temperature prior to the addition of denatured rhodanese. As a control, the aggregation of the chaperones was monitored in the reaction buffer without rhodanese. Assays were conducted in triplicate for each experiment, and at least three independent experiments were performed using batches of proteins from separate purifications. The aggregation suppression was expressed as percentage of rhodanese aggregation following normalization against assays with rhodanese alone and expressed as mean ± SEM.

### Protein-protein binding studies

Surface plasmon resonance (SPR) spectroscopy was performed using a BioRad ProteOn XPR36 optical biosensor with low immobilization GLC biosensor chip. The ProteOn phosphate buffered saline Tween-20 solution (PBST: 10 mM phosphate pH 7.4, 137 mM NaCl, 3 mM KCl, 0.005% v/v Tween-20) and ProteOn Kits (amine coupling, deactivation and post maintenance kits) were obtained from Bio-Rad Laboratories, USA. GLC chip conditioning and amine coupled ligand immobilization onto chip surfaces were performed as previously described [47]. The pH of the buffer for optimum ligand immobilisation was 4.5 for Hsp70s and the reference channel was injected with 20 mM sodium acetate (pH 4.5). A concentration of 10 μg/ml of each Hsp70 (~135 nM PfHsp70-1, ~132 nM PfHsp70-x and ~143 nM HSPA1A) was used for the immobilization. BSA and Hsj1a both at 1 μM concentration with and without ATP (1 mM) were included as controls. PBST buffer blanks with and without ATP were included either as part of the analyte runs or as separate analyte runs for double referencing. For interaction studies, a range of PFA0660w concentrations (200, 400, 600, 800 and 1000 nM) and buffer blank with 1 mM ATP were injected on the immobilized chip surface at a flow rate of 60 μl/min and a contact time of 90 s with a dissociation of 300 s. Double referencing was performed by subtraction of the blank ligand channel and the ATP buffer blank injections from PFA0660w interaction results. Each set of experiments was carried out in triplicate using at least three independent batches of purified PFA0660w. Data were analysed using ProteOn Manager (Bio-Rad, USA) and BIAevaluation 4.1.1 (GE Healthcare, UK) softwares. Global non-linear regression separate data fits were performed on the association and dissociation curves based on the built-in Langmuir association and the exponential decay models to determine the association (*k*_*a*_) and dissociation (*k*_*d*_) rate constants, and the equilibrium dissociation constants (K_D_). Qualitative interpretations of the binding assays conducted in the presence and absence of ATP were derived from inspection of the gradients for the association and dissociation curves.

## Results and Discussion

### PFA0660w was successfully purified and exists as a monomer in solution

**Fig 1:**
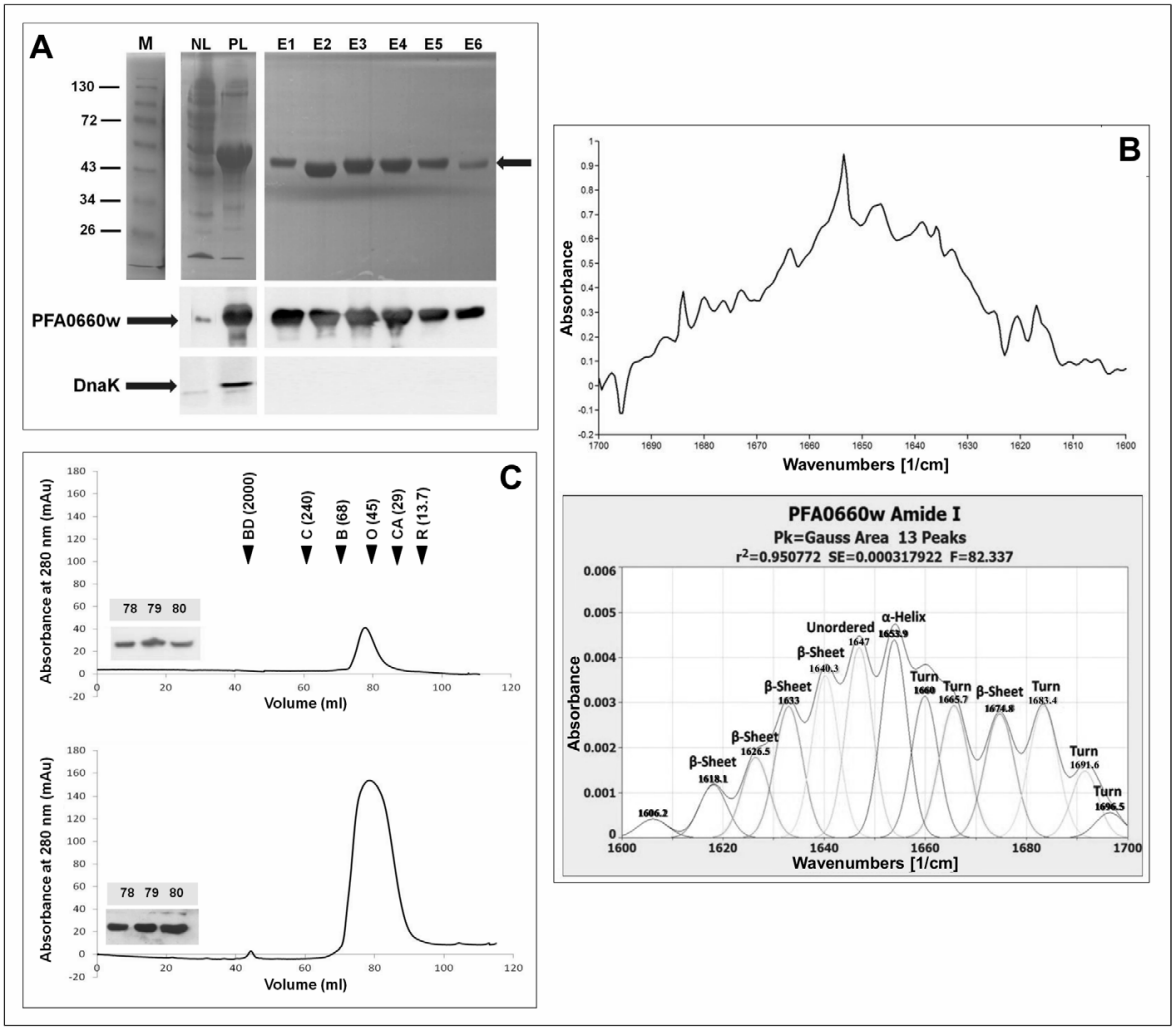
PFA0660w exists as a monomer in solution. **(A):** SDS-PAGE (**upper panel**) and western analysis (**lower panel**) of PFA0660w purification from *E*. *coli*. NL, whole cell lysis supernatant and PL, supernatant obtained from urea-solubilized pellets. E1 to E6, elution fractions following purification from PL. The arrow indicates PFA0660w on the SDS-PAGE gel. M is the protein molecular mass marker in kDa. Lower panels show western analysis with anti-His (1:5000) and anti-DnaK primary antibodies (1:5000) for confirmation of the presence of PFA0660w and lack of DnaK respectively. **(B)** The spectra of the amide I region (1600–1700 cm-^1^) of normalized native PFA0660w samples (**upper panel**) and secondary structure analysis (**lower panel**). The infrared spectra were deconvoluted and the peaks fitted with Gaussian curves. The Gaussian curves are shown as symmetrical peaks underneath the deconvoluted infrared spectra. The assigned secondary structures are shown at the top of each peak. Relative contents of the secondary structure calculated as the proportion of the peaks areas to the total area under the curve are 38.17% β-sheets, 38.82% turns, 13.03% α-helix, 12.57% unordered and 1.41% un-assigned. **(C)** Analysis of the FPLC chromatographs of PFA0660w at 0.11 mg/ml for the **upper panel** and 0.54 mg/ml for the **lower panel** with an injection volume of 2 ml. The elution volumes for the standards are indicated with black filled arrowheads and were determined by peak integration as follows: 43.79 ml for blue dextran (BD), 62.19 ml for catalase (C), 71.35 ml for BSA (B), 80.30 ml for ovalbumin (O), 87.99 ml for carbonic anhydrase (CA) and 96.25 for RNase A (R). Molecular masses in kDa are given in parenthesis and blue dextran was used to determine the void volume. Western analysis with anti-His antibody (1:5000) of elution volumes 78, 79 and 80 ml are inserted.

PFA0660w was over-expressed in *E*. *coli* M15[pREP4] cells, reaching maximum expression levels within 3 h post induction. The protein was largely insoluble and solubilization in buffer containing urea/DTT/EDTA, and refolding in DTT/EDTA/glycerol/PEG/sucrose containing buffers resulted in improved yield. Western analysis showed that PFA0660w was successfully purified and the elutions were void of DnaK (Fig 1A). The secondary structure analysis by FTIR (Fig 1B) revealed a high β-sheet content (38.17%) compared to α-helices (13.03%), which correlated well with the predicted secondary structure content from homology modelling of the C-terminal domain (data not shown) of high β-sheet content (44.3%) compared to α-helices (12.0%). Most Hsp40 proteins have been found to exist as homodimers, and dimer formation has also been shown to be important for functionality [48–52]. Therefore, the oligomeric state of PFA0660w was investigated. Size exclusion chromatography revealed a single major peak when using two different concentrations of purified PFA0660w (Fig 1C). Western analysis of the fractions confirmed that the major peak was PFA0660w. This peak eluted similarly to ovalbumin (45 kDa) (Fig 1C), indicating a predicted molecular mass for PFA0660w that was slightly higher than that predicted for a monomer (~41 kDa), but lower than that predicted for a dimer (~82 kDa). This suggested that PFA0660w existed in solution as a monomer with an extended conformation. However, since dimerization can occur upon ligand binding, it cannot be excluded that PFA0660w may form dimers or heterodimers, for instance with PFE0055c within the J-dots *in vivo* when interacting with its partner proteins and substrates.

### PFA0660w selectively stimulated the ATPase activity of PfHsp70-x

**Fig 2:**
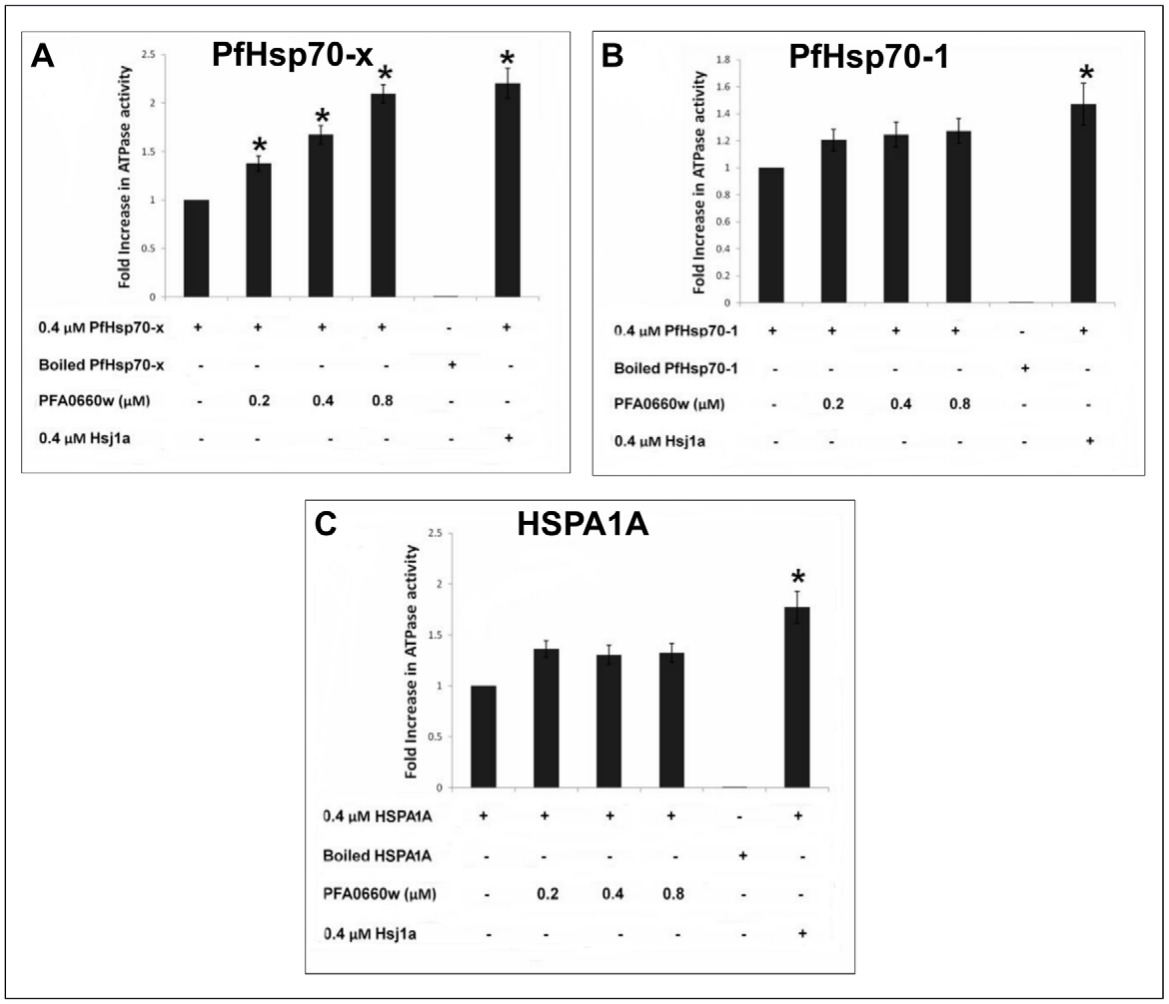
PFA0660w stimulates the ATPase activity of PfHsp70-x. The bar graphs show the basal and PFA0660w-stimulated ATPase activities of PfHsp70-x (**A**), PfHsp70-1 (**B**) and HSPA1A (**C**) expressed as mean ± SEM. Each set of bar graphs represents the fold increase in the ATPase activity of the Hsp70 alone (0.4 μM) or in combination with sub-molar (0.2 μM), equimolar (0.4 μM) and molar excess (0.8 μM) concentrations of PFA0660w or equimolar (0.4 μM) concentration of Hsj1a. The boiled samples of Hsp70s and native sample of Hsj1a serve as negative and positive controls respectively. All samples were corrected for spontaneous ATP hydrolysis before normalization to obtain fold increase. Error bars are indicated on each bar and * indicates statistical significance at P<0.05 relative to basal ATPase value for respective chaperone using Student T-test. constituents that were either included or omitted from the reaction medium are indicated by (+) or (-) sign, respectively. Shown here are the combined data from three independent experiments performed in triplicate using at least three batches of independently purified proteins for each experiment.

Given that PFA0660w has been shown to be physically associated with PfHsp70-x in J-dots, we tested its ability to modulate the ATPase activities of PfHsp70-x, HSPA1A (another potential partner) and PfHsp70-1 (an unlikely partner). The basal specific ATPase activities for PfHsp70-x, PfHsp70-1 and HSPA1A were comparable, with values of 12.79 ± 0.13, 9.15 ± 0.26 and 8.40 ± 0.16 nmol Pi/min/mg, respectively. In the presence of PFA0660w, the results showed a concentration-dependent and a statistically significant (P<0.05) increase in the stimulation of the ATPase activity of PfHsp70-x while the stimulation of PfHsp70-1 and HSPA1A was neither concentration-dependent nor significant (Fig 2A–2C). A previously used positive control Hsp40, Hsj1a [14,23,32], significantly stimulated the ATPase activity of each Hsp70 as expected (Fig 2A–2C). Neither Hsj1a nor PFA0660w displayed any ATPase activity (data not shown). The ATPase assays showed that all of the Hsp70s were functional as their basal activities could be stimulated by the control Hsj1a. In contrast to Hsj1a, PFA0660w only stimulated the ATPase activity of PfHsp70-x, suggesting functional specificity for the exported Hsp70.

### PFA0660w is able to suppress the aggregation of rhodanese

**Fig 3:**
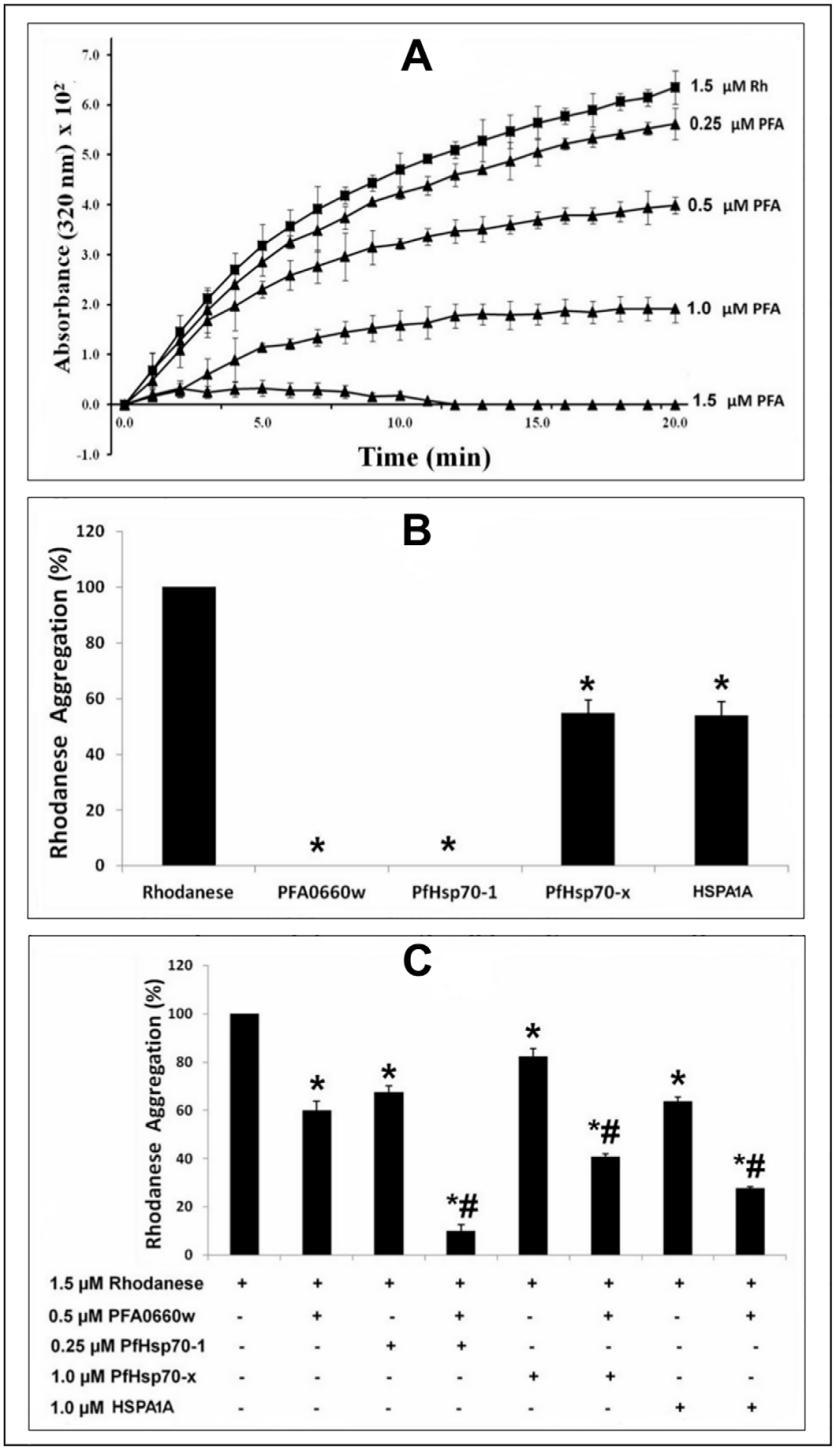
PFA0660w suppresses rhodanese aggregation. Rhodanese aggregation suppression assays were performed for 20 min at room temperature. (**A**) The curves of absorbance at 320 nm (x 10^2^) versus time (min) showing aggregation of rhodanese alone (1.5 μM Rh, *filled black squares*) or in the presence of a range of concentrations (*filled triangles*) of recombinant PFA0660w (PFA). PFA0660w concentrations are shown at the end of each progress curve. The error bars are indicated for each plotted data. The absorbance values were multiplied by 10^2^ so that the calculated errors can be clearly seen on the graph. Twenty time points were used to plot the progress curves. PFA0660w showed a concentration-dependent suppression of rhodanese aggregation. (**B**) Bar graphs showing the comparison of the effects of 1.5 μM of PFA0660w, PfHsp70-1, PfHsp70-x and HSPA1A on rhodanese aggregation. Both PFA0660w and PfHsp70-1 produced a complete suppression at 1.5 μM. (**C**) The effect of PFA0660w (0.5 μM) on rhodanase aggregation suppression activity of PfHsp70-x (1.0 μM), PfHsp70-1 (0.25 μM) and HSPAIA (1.0 μM) respectively. BSA (1.5 μM) was used as a control, and the rhodanese aggregation in the presence of BSA was set as 100% (not shown). Error bars are indicated. An * or # indicates statistical significance at P<0.05 when compared to rhodanese or to both Hsp70s and PFA0660w respectively, using Student T-test. In **C**, constituents that were either included or omitted from the reaction medium are indicated by (+) or (-) sign, respectively. Shown here are the combined data from three independent experiments performed in triplicate using at least three batches of independently purified proteins for each experiment.

Since PFA0660w could specifically stimulate the ATPase activity of PfHsp70-x, we next tested its ability suppress protein aggregation alone and together with PfHsp70-x, HSPA1A and PfHsp70-1. PFA0660w produced a concentration-dependent decrease in the aggregation of chemically denatured rhodanese when used on its own (Fig 3A). This indicated that PFA0660w possessed independent protein aggregation suppression activity. PFA0660w and PfHsp70-1 completely suppressed the aggregation of rhodanese at 1.5 μM, while the same concentration of PfHsp70-x and HSPA1A produced a 45.10 ± 1.32% and 45.99 ± 0.68% reduction of aggregation respectively (Fig 3B). These data suggested that PFA0660w suppresses the aggregation of rhodanese more effectively than PfHsp70-x and HSPA1A. The effect of PFA0660w (0.5 μM) on the aggregation suppression activities of PfHsp70-1 (0.25 μM), PfHsp70-x (1 μM) and HSPAIA (1 μM) was further determined. The concentrations of the Hsp70s used in the assay were those that produced a significant reduction in rhodanese aggregation but with aggregation still greater than 50%. BSA (1.5 μM) was used as a control and did not produce any effect on the aggregation of rhodanese (data not shown). While PFA0660w (0.5 μM) produced an additive effect on the protein aggregation suppression activities of each Hsp70, no major stimulatory effect was observed (Fig 3C). PFA0660w appears to be relatively efficient at the suppression of protein aggregation, with activity higher than that reported for other Hsp40 proteins [46,53]. PFA0660w may function as both an independent chaperone or ‘holdase’ and a co-chaperone passing unfolded proteins onto PfHsp70-x and stimulating its ATPase activity.

### PFA0660w binding to PfHsp70-x is consistent with a co-chaperone role

**Fig 4:**
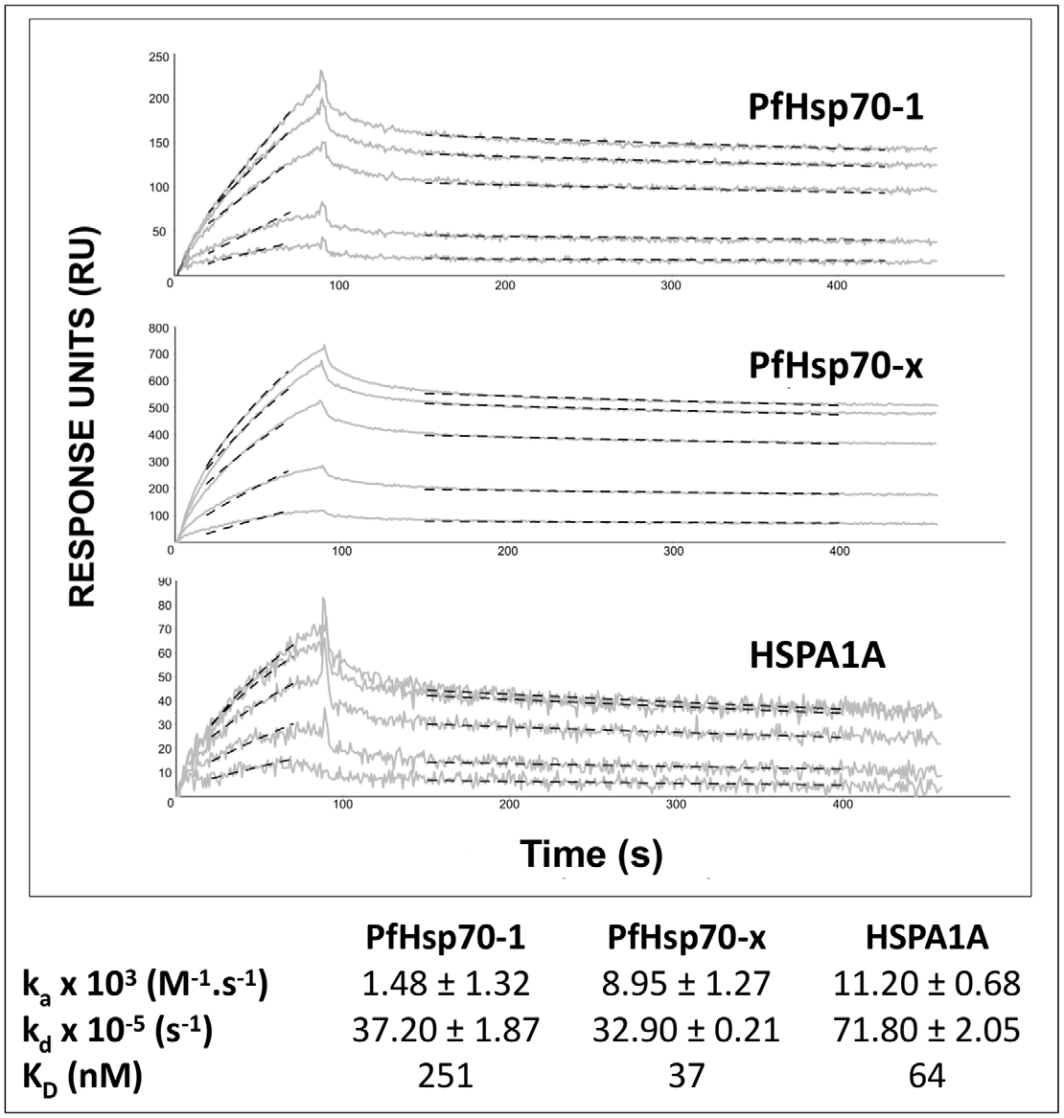
Kinetics of the Interaction of PFA0660w with PfHsp70-x, PfHsp70-1 and HSPA1A. Shown are the representative concentration dependent SPR sensorgrams of increasing concentrations of PFA0660w (200–1000 nM) passed over the immobilised Hsp70s in the presence of 1 mM ATP. Solid grey and dotted black lines indicate collected sensorgram data and data fits respectively. Regions selected for data fitting were 20 s after analyte injection and 20 s before end of injection for association phase. Region selected was based on dy/dx derivitization of data. The exponential decay model was fit to the stable portion of the dissociation phase. The lower panel shows the kinetic rate constants from separate association and dissociation curve non-linear regression fits (mean ± standard deviation from multiple replicates; n = 3), and equilibrium dissociation constants (K_*D*_ values) calculated from the kinetic rate constants. The interaction assays for each replicate were generated with freshly prepared analytes and ATP. All data were double referenced using buffer blanks and blank channel.

**Fig 5:**
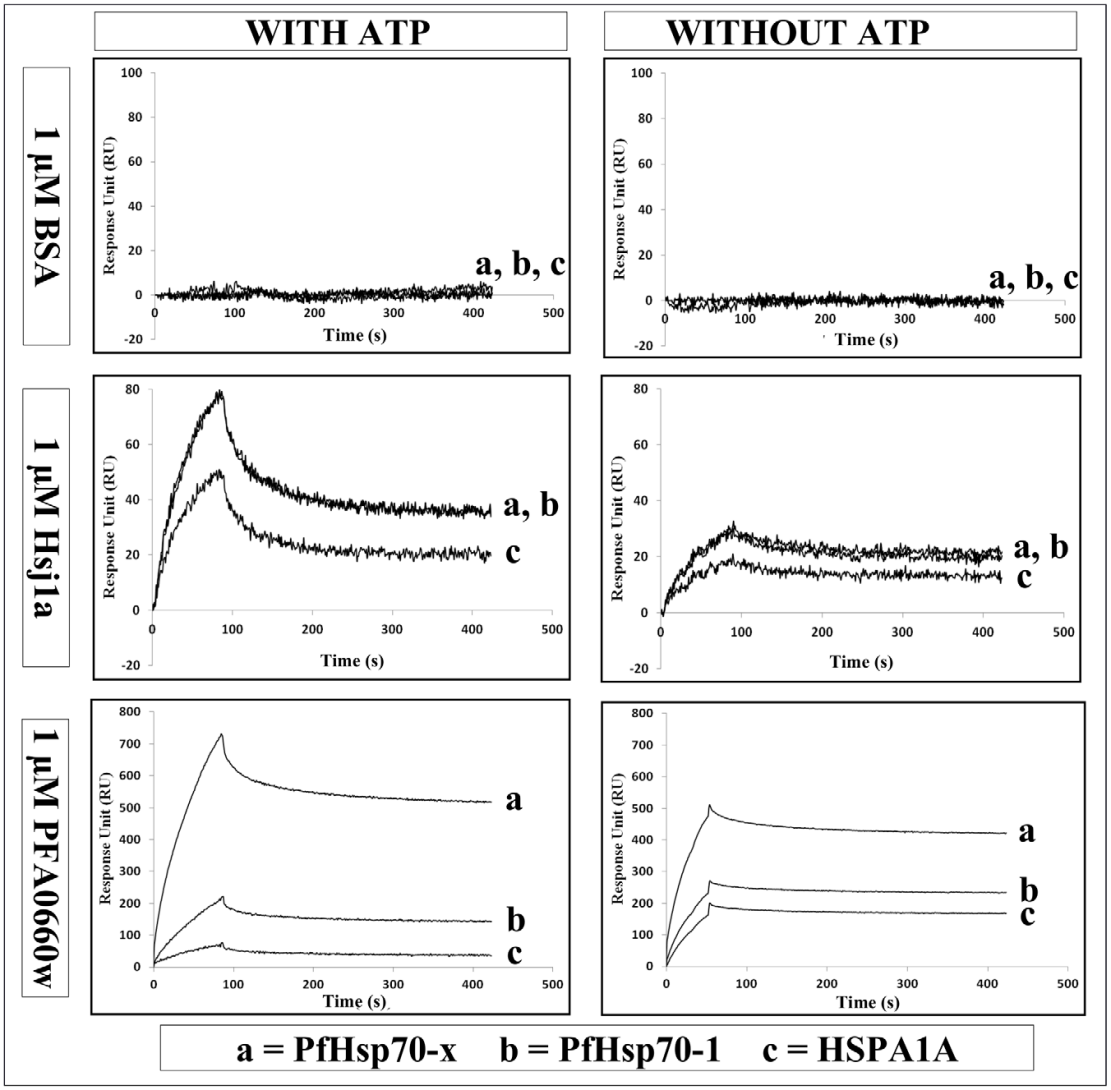
The effects of ATP on the interaction of PFA0660w with PfHsp70-x, PfHsp70-1 and HSPA1A. The effects of ATP on the interaction of BSA, Hsj1a and PFA0660w with Hsp70s were tested using SPR spectroscopy. The analysis was performed by passing BSA, Hsj1a or PFA0660w (1 μM) over the immobilised Hsp70s in the presence and absence of 1 mM ATP. BSA and Hsj1a served as controls. The interaction assays were performed in triplicate and repeated at least three times with freshly prepared analytes and ATP. All data were double referenced using buffer blanks (with or without ATP) and blank channel. Shown here are representative sensograms for the interaction of BSA (**upper panel**), Hsj1a (**middle panel**) and PFA0660w (**lower panel**) with PfHsp70-x (a), PfHsp70-1 (b) and HSPA1A (c).

The functional assays implied that PFA0660w interacted specifically and directly with PfHsp70-x as a co-chaperone, and so we used SPR spectroscopy to further assess direct binding. These binding assays were performed in presence of 1 mM ATP to abrogate any substrate/chaperone interactions [54,55]. The kinetics of PFA0660w binding to immobilized PfHsp70-1, PfHsp70-x and HSPA1A suggested there was direct binding with slightly different association and dissociation rate constants (Fig 4). The K_*D*_ values suggested that the affinities of PFA0660w for PfHsp70-x (37 nM) and HSPA1A (64 nM) were similar, and greater than its affinity for PfHsp70-1 (251 nM). However, since Hsp70s undergo conformational changes in the presence of ATP due to their ATPase activity, conclusions from comparison of these affinities should be considered preliminary. To further explore the co-chaperone/chaperone nature of the associations, the interaction of PFA0660w with the Hsp70s was monitored by SPR spectroscopy in the presence (substrate interactions abrogated) and absence (substrate interactions possible) of ATP (Fig 5). A BSA negative control showed no interaction with the Hsp70s either in the presence or absence of ATP, whilst the positive control Hsj1a showed increased binding to all the Hsp70s in the presence of ATP (Fig 5). This was consistent with the fact that it functioned as a co-chaperone and stimulated the ATPase activity of all the Hsp70s (Fig 2). On the other hand, PFA0660w only showed increased binding to PfHsp70-x in the presence of ATP (Fig 5), the only chaperone whose ATPase activity it was able to stimulate (Fig 2). Interestingly, the binding of PFA0660w to PfHsp70-1 and HSPA1A was reduced in the presence of ATP compared to without ATP (Fig 5). Overall, these data suggested that the interaction of PFA0660w with PfHsp70-x most likely represented a co-chaperone/chaperone interaction.

## Conclusion

We have provided the first biochemical evidence for a specific functional co-chaperone interaction between the exported malarial PFA0060w and PfHsp70-x. PFA0660w was able to specifically stimulate the ATPase activity of PfHsp70-x and work additively with it in suppressing protein aggregation. It also showed considerable protein aggregation suppression activity alone, suggesting that it could potentially act independently as a chaperone. Protein binding studies in the presence and absence of ATP suggested that the interaction of PFA0660w with PfHsp70-x most likely represented a co-chaperone/chaperone interaction. These findings are consistent with, and support, the proposed role of PfHsp70-x and PFA0660w in parasite protein trafficking and folding in the infected erythrocyte cytosol. Further studies are underway to determine the molecular basis for the specificity of this interaction, and to identify small-molecule inhibitors capable of disrupting the interaction.
